# Association of Cytokeratin 5 and Claudin 3 expression with *BRCA1* and *BRCA2* germline mutations in women with early breast cancer

**DOI:** 10.1186/s12885-019-5908-6

**Published:** 2019-07-15

**Authors:** Sabine Danzinger, Yen Yen Tan, Margaretha Rudas, Marie-Theres Kastner, Sigrid Weingartshofer, Daniela Muhr, Christian F. Singer

**Affiliations:** 1 Department of Obstetrics and Gynecology, Comprehensive Cancer Center, Medical University of Vienna, 1090 Vienna, Austria; 2 Department of Pathology, Comprehensive Cancer Center, Medical University of Vienna, 1090 Vienna, Austria

**Keywords:** Familial breast cancer, *BRCA1*, *BRCA2*, Tissue microarray, Immunohistochemistry, Claudin

## Abstract

**Background:**

It is important to identify biomarkers associated with *BRCA* mutation in women with early breast cancer (BC) to improve early identification of mutation carriers. Thus, in this study, we examined the protein expression of claudin (CLDN) 3, CLDN4, CLDN7, and E-cadherin. Moreover, we analyzed additional histopathological variables and their associations in familial BC.

**Methods:**

Immunohistochemical analysis for CLDNs and E-cadherin was performed on 237 BC cases of three different subsets of BC tumors: 62 from *BRCA1* mutation carriers*,* 59 from *BRCA2* mutation carriers, and 116 tumors from patients with *BRCA* wild type (WT) as controls. Histopathological data were also analyzed in the different subgroups. Logistic regression and receiver operation characteristic (ROC) curve were conducted to investigate factors associated with *BRCA* tumors.

**Results:**

Expression of CLDN3 positively correlated with *BRCA*-mutated BC. CLDN3 was expressed in 58% of *BRCA1*-mutated tumors compared to only 7% in *BRCA2*-mutated tumors (*p* < 0.001) and 1% in WT tumors (*p* < 0.001). CK5 and CK14 expression were also more likely to arise in *BRCA1* tumors (44 and 16%, respectively) than in the control group (8 and 4%) (*p* < 0.001, *p* = 0.012, respectively). We also found a significantly higher proportion of CK5+ among *BRCA1* tumors (44%) in comparison with *BRCA2*-related BC (8%) (*p* < 0.001). In addition, there was a significant difference between both groups regarding CK14: positive expression in 16 and 5%, respectively (*p* = 0.030). CK5 and CK14 did not differ between the *BRCA2* group and the WT tumors significantly. In a multivariate regression model, expression of CK5 (Odds ratio (OR): 6.46; 95% confidence interval (CI): 1.52–27.43; *p* = 0.011), and CLDN3 (OR: 200.48; 95% CI: 21.52–1867.61; *p* < 0.001) were associated with *BRCA1* mutation status.

**Conclusions:**

Our data suggests that CLDN3, CK5, and CK14 in combination with ER, PR and HER2 are associated with *BRCA1* mutation status.

**Electronic supplementary material:**

The online version of this article (10.1186/s12885-019-5908-6) contains supplementary material, which is available to authorized users.

## Background

Breast cancer (BC) is the leading cancer type among women in the world [1]. Familial BC, representing 5–7% of all BC, are hereditary and are associated with inherited gene mutations [2]. Approximately 25% of familial BC are due to germline mutations in the *BRCA1* and *BRCA2* genes, which are located on chromosome 17 and 13, respectively [2–4]. The average cumulative BC risk in *BRCA1* mutation carriers by age 70 is 57–65%, whereas the cumulative BC risk in patients with *BRCA2* mutation is 45–49% [5, 6].

*BRCA1*-associated tumors show a more aggressive phenotype, the majority of these tumors are invasive ductal adenocarcionomas (74%) and are poorly differentiated (high histological grade) [7–13]. More than 75% of *BRCA1*-mutated tumors are triple-negative, have a basal-like phenotype, or both [2, 7, 10, 14–20]. Triple-negative BC is characterized by lack of expression of hormone receptors (ie. estrogen receptor (ER) and progesterone receptor (PR)), and human epidermal growth factor receptor 2 (HER2) [21]. Basal-like BC, a subtype of triple-negative BC, can be characterized by the expression of basal cytokeratins (CK) (such as CK5/6, CK14) and epidermal growth factor receptor (EGFR), among others [14, 16, 22–28].

Claudin-low is another subtype of triple-negative BC, and can be characterized by low expression of claudin (CLDN) 3, CLDN4, CLDN7, and E-cadherin. The majority of claudin-low tumors have a poor prognosis [29–31]. CLDNs are structural and functional components of tight junctions which provide cell-cell adhesion in epithelial to endothelial cells [32]. There are at least 24 different CLDNs existing in humans, the expression of each seems to be tissue specific [33]. E-cadherin is one of the most important molecules in cell-cell adhesion in epithelial tissues [34]. Loss of intercellular adhesion by E-cadherin correlates with increased invasiveness and metastasis of tumors [34–39].

On the contrary, *BRCA2*-mutated tumors are more heterogeneous. The immunophenotype of *BRCA2*-associated tumors is very similar to sporadic BC. They are frequently characterized by low/intermediate histological grade. They often show no or low expression of HER2 and are often positive for ER and PR than in *BRCA1*-related tumors. Furthermore, *BRCA2*-mutated tumors do not express CK5, CK6 and CK14 [2, 7, 10, 13, 17, 40–44].

In this study, we analyzed clinicohistopathological features which are already associated with *BRCA1/2* tumors (ER, PR, HER2, CK5 and 14, EGFR, among others). In addition, we selected three important CLDNs in BC, ie. CLDN3/4/7, and E-cadherin, which are used for characterization of the claudin-low subtype. We aim to define the expression profiles of these biomarkers in *BRCA1* BC and compare these with *BRCA2* and *BRCA* WT patients to improve early identification of mutation carriers.

We presented the study as an abstract at the 15th St. Gallen International Breast Cancer Conference in Vienna, Austria. [Danzinger S et al.: Intratumoral Cytokeratin 5 and Claudin 3 protein expression predicts for the presence of BRCA1 germline mutation in women with early breast cancer. The Breast 2017, 32 (Suppl 1):S22–77.]

## Methods

### Study population

A total of 242 BC tissue microarrays (TMA) were obtained from the Kathleen Cuningham Foundation Consortium for research into Familial Breast cancer (kConFab) [http://www.kconfab.org]. We evaluated one case per patient, and one core per case. The core diameter was 0.6 mm. Three BC cases were excluded due to *TP53* (*n* = 1) and *PALP2* (*n* = 2) mutation status. We also eliminated two cases of ductal carcinoma in situ. Our analysis was therefore based on 237 BC tumors where 62 tumors originated from *BRCA1* carriers, 59 tumors were from *BRCA2* carriers, and we obtained the remaining 116 from *BRCA* WT patients. The *BRCA* WT subgroup served as controls in our study. The control group consists of tumors from non *BRCA1/2* mutation carriers. We used these tumors from consecutive BC patients with familial history. *BRCA* testing and analysis are described in the Supplemental (Additional file 1). Clinicopathological information collected included age at diagnosis, tumor size, tumor morphology, tumor grade, ER, PR, HER2, CK5 and 14, and EGFR.

Immunohistochemical analysis for ER, PR, HER2, CK5 and 14, and EGFR.

Immunohistochemical staining of the samples was performed as described in our previous study [45]. The following antibodies were used: clone SP1 against ER (prediluted, 790–4296, Ventana Medical Systems Inc., Tucson, AZ, USA), clone 1E2 against PR (790–4325, Ventana Medical Systems Inc.), clone 4B5 against HER2 (prediluted, 800–2996, Ventana Medical Systems Inc.), clone EP1601Y against CK5 (305R-16, Cell Marque Corporation, Rocklin, CA, USA), clone LL002 against CK14 (LL002-L-CE, Leica, Novocastra, Newcastle upon Tyne, United Kingdom), and clone 31G7 against EGFR (28–0005, Zymed, South San Francisco, CA, USA).

ER and PR were considered positive if there were ≥ 1% tumor nuclei stained according to the American Society of Clinical Oncology/College of American Pathologists (ASCO/CAP) Guideline [46]. HER2-positivity was defined by staining of > 10% of tumor cells as proposed by the update of the American Society of Clinical Oncology/College of American Pathologists (ASCO/CAP) clinical practice guideline. Staining of HER2 should also be strong and circumferentially membranous [47]. CK5, CK14, and EGFR were regarded as positive if any cytoplasmic and/or membranous staining was seen in the tumor cells [33].

Immunohistochemical analysis for CLDN3/4/7 and E-cadherin.

Immunohistochemical staining for CLDN3, CLDN4, CLDN7, and E-cadherin was performed according to the protocol of our previous study [45]. For immunohistochemistry of paraffin-embedded sections (5 μm), we used the ultraView Universal DAB Detection Kit (5269806001, 760–500, Ventana Medical Systems Inc., Tucson, AZ, USA), and an automated immunostainer system (Ventana Benchmark, Ventana Medical Systems Inc.). Tissues were processed with high-temperature technique for 30 min (CLDN3 and CLDN7) or 60 min (CLDN4, E-cadherin) and incubated with antibodies.

We used the following antibodies for staining the tissue sections: Rabbbit anti-Claudin-3 against CLDN3 (32 min; 34–1700, Invitrogen, Thermo Fisher Scientific, Rockford, IL, USA), Mouse anti-Claudin-4 (monoclonal) against CLDN4 (64 min; 32–9400, Invitrogen), Rabbit anti-Claudin-7 against CLDN7 (32 min; 34–9100, Invitrogen), and anti-E-Cadherin (36) Mouse Monoclonal against E-cadherin (16 min; 790–4497, Ventana Medical Systems Inc.). The antibody concentrations for CLDN3, CLDN4, and CLDN7 were in the range of 2–3 μg/ml, the antibody concentration for E-cadherin was 0.314 μg/ml.

We used hydrogen peroxide and 3,3’-diaminobenzidine-tetrahydrochloride for visualization of the reaction. The slides were counterstained with haemalaun and exposed to a bluing reagent for different times. We used colon (CLDN3, E-cadherin) and breast tissue (CLDN7) as positive controls. CLDN4 was overexpressed in ovarian cancer.

Immunohistochemical staining was centrally reviewed by an experienced pathologist (MR) to ensure comparable results. The stained sections were visualized on an Olympus BX50 microscope (Olympus Corporation, Tokyo, Japan). The imaging software cell^P (Olympus Corporation) was used for taking pictures of the slides. The tumor samples were evaluated by differentiation between positive and negative staining. Only membranous staining was classified as positive. There is no standard for assessing the CLDN expression [48]. Positive expression of CLDN3, CLDN4, and CLDN7 was defined by any detectable staining in the membrane of the tumor cell. E-cadherin was regarded positive if any staining was observed. Thus, complete absence of any membranous E-cadherin immunoreactivity was considered as E-cadherin-negative [49].

### Statistical methods

Descriptive statistics were performed to determine the characteristics of our study sample, which comprise three groups: *BRCA1, BRCA2,* and *BRCA* WT. ANOVA test and student’s t-test were used to compare the mean age at diagnosis between two groups. Chi-square and Fisher’s Exact (for smaller sample size) tests were used to compare the proportions of clinicohistopathological (categorical) parameters. To further determine the relationship between clinicohistopathological parameters with mutation status, Spearman’s correlation analysis was performed. Logistic regression was conducted to identify independent factors associated with *BRCA1* (and *BRCA2*) mutation status. Associations were summarized using the odds ratio (OR) and corresponding 95% confidence interval (CI) derived from the model estimates. The receiver operation characteristic (ROC) curves were constructed for the prediction of *BRCA1* mutation status. The predictive ability of each model was summarized by the area under the curve (AUC), and optimal models were classified as those that yielded the highest AUC in the ROC analysis. We excluded all unknowns/undetermined values from analysis. Statistical significance was considered as *p* < 0.05 (2-tailed). We performed all statistical analyses using SPSS (v. 23.0 (SPSS Inc., Chicago, IL, USA)).

## Results

**Fig. 1:**
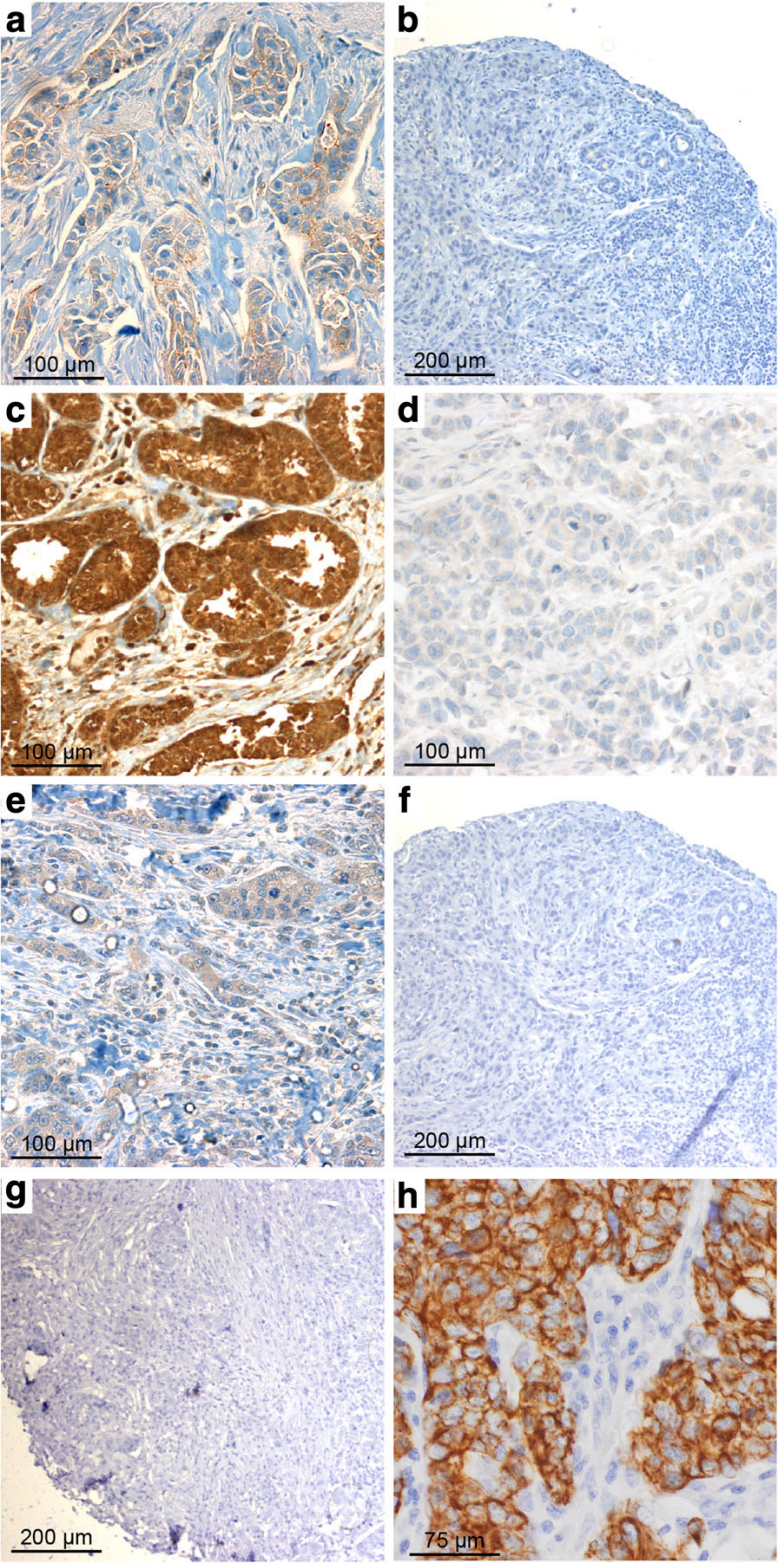
Claudin (CLDN) 3. Positive expression in *BRCA1* breast cancer (BC) (**a**) and negative control (isotypic antibody) (**b**). Positive CLDN4 expression in *BRCA1*-mutated BC (**c**), negative control of CLDN4 (isotypic antibody) (**d**). Positive CLDN7 in *BRCA2* BC (**e**), negative CLDN7 in *BRCA1*-related tumor (**f**), and negative control of CLDN7 (isotypic antibody) (**g**). Positive CK5 staining in *BRCA1* BC (**h**)

**Fig. 2:**
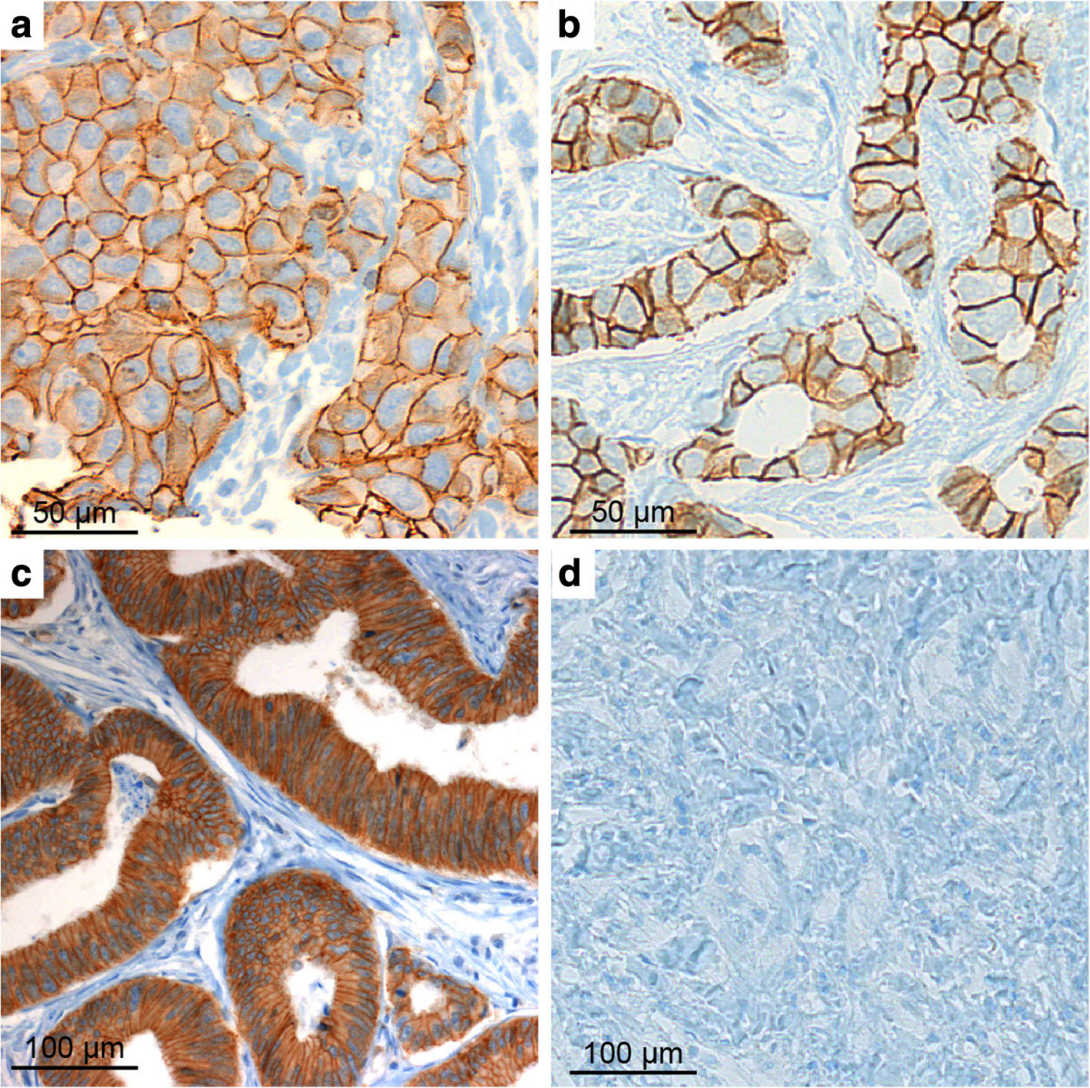
E-cadherin. Positive staining in *BRCA1*-mutated (**a**) and *BRCA* wild type breast cancer (**b**). Positive benign epithelium (colon) as positive control (**c**). Negative staining in the wild type group (**d**)

The characteristics of the patients are summarized in Table 1. Immunohistochemical staining of CLDN3, CLDN4, and CLDN7, and E-cadherin is shown in Fig. 1 and Fig. 2. The tumor size of most of the tumors in all of the three groups (45% of *BRCA1*, 53% of *BRCA2*, and 63% of WT tumors) was < 2 cm. The histological type is dominated by invasive ductal carcinoma in all groups (72–90%). Grade 3 was the most common tumor grade in *BRCA1* tumors (65%), followed by *BRCA2* (44%) tumors, compared to only 27% of *BRCA* WT tumors (*p* < 0.001, *p* = 0.006, respectively).

**Table 1:** Characteristics of tumors (WT = *BRCA* wild type breast cancer, BRCA1, BRCA2 = breast cancer in *BRCA1/BRCA2* mutation carriers, ER = estrogen receptor, PR = progesterone receptor, HER2 = human epidermal growth factor receptor 2, CK = cytokeratin, EGFR = epidermal growth factor receptor, CLDN = claudin)

Characteristics		WT (*N* = 116)		BRCA1 (*N* = 62)		BRCA2 (*N* = 59)		WT v BRCA1	*p*-value (Chi = square or Fisher’s test (if cells < 5))*		
		N	Col %	N	Col %	N	Col %		WT v BRCA2	BRCA1 v BRCA2	WT v BRCA1/2
Age at diagnosis	<50y	79	68	47	76	38	64	0.282	0.623	0.170	0.721
	> = 50y	37	32	15	24	21	36				
Tumor grade	1	29	25	1	2	4	7	<.001	0.006	0.021	<.001
	2	37	32	12	19	22	37				
	3	31	27	40	65	26	44				
	Unknown	19	16	9	15	9	15				
Tumor size	< 2 cm	73	63	28	45	31	53	0.152	0.272	0.411	0.147
	2-5 cm	35	30	26	42	20	34				
	> 5 cm	2	2	1	2	3	5				
	Unknown	6	5	7	11	7	12				
Tumor morphology	Invasive ductal carcinoma	83	72	56	90	47	80	0.027	0.327	0.072	0.068
	Invasive lobular carcinoma	8	7	0	0	5	8				
	Invasive ductal+lobular carcinoma	6	5	0	0	2	3				
	Carcinoma – undefined	4	3	2	3	3	5				
	Other	15	13	4	6	2	3				
ER	Negative	28	24	47	76	13	22	<.001	0.448	<.001	<.001
	Positive	78	67	14	23	45	76				
	Unknown	10	9	1	2	3	5				
PR	Negative	42	36	49	79	23	39	<.001	0.89	<.001	0.001
	Positive	68	59	11	18	34	58				
	Unknown	6	5	2	3	4	7				
HER2	Negative	77	66	43	69	22	37	0.887	<.001	<.001	0.003
	Positive	22	19	13	21	32	54				
	Unknown	17	15	6	10	7	12				
CK5	Negative	78	67	28	45	44	75	<.001	0.702	<.001	0.001
	Positive	9	8	27	44	5	8				
	Unknown	29	25	7	11	12	20				
CK14	Negative	89	77	45	73	49	83	0.012	0.999	0.030	0.129
	Positive	5	4	10	16	3	5				
	Unknown	22	19	7	11	9	15				
EGFR	Negative	76	66	52	84	52	88	0.585	0.052	0.209	0.122
	Positive	10	9	5	8	1	2				
	Unknown	30	26	5	8	8	14				
CLDN3	Negative	107	92	24	39	50	85	<.001	0.039	<.001	<.001
	Positive	1	1	36	58	4	7				
	Unknown	8	7	2	3	7	12				
CLDN4	Negative	6	5	6	10	0	0	0.423	0.094	0.999	0.059
	Positive	100	86	56	90	58	98				
	Unknown	10	9	5	8	3	5				
CLDN7	Negative	109	94	62	100	57	97	n/a	0.339	0.475	0.999
	Positive	0	0	0	0	1	2				
	Unknown	7	6	0	0	3	5				
E-cadherin	Negative	10	9	9	15	16	27	0.296	0.002	0.081	0.016
	Positive	89	77	48	77	40	68				
	Unknown	17	15	5	8	5	8				

*unknown/undetermined values are excluded from analysis

Negative expression of ER and PR was significantly more common among *BRCA1* mutation carriers (76 and 79%, respetively) compared to the *BRCA2* group (22 and 39%, respectively) (both *p* < 0.001) and in comparison with the WT subgroup (24 and 36%, respectively) (both *p* < 0.001). HER2 was positive in 54% of *BRCA2* versus 19% of WT tumors (*p* < 0.001) and versus 21% of *BRCA1*-mutated tumors (*p* < 0.001). Positive HER2 was found to be associated with a *BRCA2* mutation compared to the WT.

Tumors with expression of CK5 and CK14 were more likely to arise in *BRCA1* (44 and 16%, respectively) than in the control group (8 and 4%) (*p* < 0.001, *p* = 0.012, respectively). We also found a significantly higher proportion of positive CK5 expression among *BRCA1* tumors (44%) in comparison with *BRCA2*-related BC (8%) (*p* < 0.001). CK14 was positive in 16% of *BRCA1* versus 5% of *BRCA2* tumors (*p* = 0.030). Furthermore, EGFR negatively correlated with the *BRCA2* mutation status in comparison with *BRCA* WT with statistical significance.

Thirty-six of 41 CLDN3-positive cases had a *BRCA1* mutation, and 40 of 41 such cases have a mutation in either *BRCA1* or *BRCA2.* CLDN3 was positively correlated with *BRCA1*-mutated BC. Positive CLDN3 was found in 58% of *BRCA1*-mutated tumors compared to only 7% of *BRCA2*-mutated tumors (*p* < 0.001) and 1% of WT tumors (*p* < 0.001). Positive CLDN3 was also significantly more frequent in the *BRCA2* in comparison with the WT group (*p* = 0.039) (Table 1). A positive correlation was observed between CLDN3 with tumor grade and CK5. In contrast, CLDN3 negatively correlated with ER and PR.

E-cadherin expression was significantly different in proportion between tumors of *BRCA* WT and *BRCA2* mutation (77% versus 68%, respectively, *p* = 0.002). With regard to CLDN4 and CLDN7, there were no significant differences observed among the groups. Positive CLDN4 was very common (86–98%), but CLDN7 was rarely positive (0–2%).

In an univariate analysis of clinicopathological factors associated with *BRCA1*-mutated BC versus the WT subtype, tumor grade, ER, PR, and expression of CK5, CK14, and CLDN3 were found to be independent parameters. However, when these features were put into a multivariate regression model and adjusted with age, only CK5+ (Odds ratio (OR): 6.46; 95% Confidence interval (CI): 1.52–27.43; *p* = 0.011), and CLDN3+ (OR: 200.48; 95% CI: 21.52–1867.61; *p* < 0.001) they revealed to be associated with *BRCA1* mutation status (Table 2).

**Table 2:** Logistic regression for *BRCA1* versus WT and *BRCA2* versus WT: univariate and multivariate analysis

Characteristics		Univariate LR OR (95%CI)	*p*-value	Multivariate LR^a^ OR (95%CI)	*p*-value	Univariate LR OR (95%CI)	*p*-value	Multivariate LR^a^ OR (95%CI)	*p*-value
		*BRCA1* vs *WT*				*BRCA2* vs *WT*			
Age at diagnosis (years)	<50y	1.0				1.0			
	≥50y	0.68 (0.34–1.37)	0.283			1.18 (0.61–2.28)	0.624		
Tumor grade	1 + 2	1.0				1.0		1.0	
	3	6.55 (3.07–13.97)	<.001			2.13 (1.06–4.29)	0.034	2.41 (1.02–5.69)	0.045
Tumor size	< 2 cm	1.0				1.0		–	
	> = 2 cm	1.90 (.98–3.68)	0.056			1.51 (0.77–2.96)	0.227	–	
ER	Negative	1.0				1.0		–	
	Positive	.11 (.05–.22)	<.001	0.46 (0.09–2.66)	0.400	1.35 (0.62–2.91)	0.449	–	
PR	Negative	1.0				1.0		–	
	Positive	0.14 (0.07–0.30)	<.001	0.35 (0.07–1.87)	0.354	0.96 (0.49–1.85)	0.890	–	
HER2	Negative	1.0				1.0		1.0	
	Positive	1.06 (.49–2.31)	0.887	1.03 (0.24–4.48)	0.970	5.33 (2.58–11.03)	<.001	5.21 (2.18–12.45)	<.001
CK5	Negative	1.0				1.0		–	
	Positive	8.36 (3.50–19.93)	<.001	6.46 (1.52–27.43)	0.011	0.79 (0.23–2.71)	0.705	–	
CK14	Negative	1.0				1.0		–	
	Positive	3.96 (1.28–12.27)	0.017			0.73 (0.14–3.89)	0.709	–	
EGFR	Negative	1.0				1.0			
	Positive	0.73 (.24–2.26)	0.586			0.15 (0.02–1.20)	0.074		
CLDN3	Negative	1.0				1.0			
	Positive	160.50 (20.96–1229.06)	<.001	200.48 (21.52–1867.61)	<.001	8.92 (0.97–81.90)	0.053		
CLDN4	Negative	1.0		–		–		–	
	Positive	3.36 (0.39–28.62)	0.267	–		–		–	
CLDN7	Negative	–		–		–		–	
	Positive	–		–		–		–	
E-cadherin	Negative	1.0		–		1.0		1.0	
	Positive	0.60 (0.23–1.58)	0.299	–		0.27 (0.11–0.64)	0.003	0.33 (0.11–0.98)	0.046

^a^adjusted for age at diagnosis

For *BRCA2* BC versus WT, univariate analysis showed that tumor grade, the expression of HER2, and E-cadherin were independent markers of *BRCA2* status. In a multivariate regression model, the expression of HER2 (OR: 5.21; 95% CI: 2.18–12.45; *p* < 0.001) showed an association with *BRCA2* mutation status (Table 2).

For *BRCA2* versus *BRCA1* tumors, univariate analysis showed that tumor grade, ER, PR, HER2, CK5, CK14, and CLDN3 were independent parameters of *BRCA2* status. However, in a multivariate model, all parameters were not significantly associated with the mutation status, except for CLDN3. Positive CLDN3 in *BRCA2*-related tumors has an OR of 0.05 (95% CI: 0.01–0.26; *p* < 0.001) with reference to *BRCA1* tumors. Thus, negative CLDN3 was found in 85% of *BRCA2*-related tumors versus 39% of *BRCA1* BC (*p* < 0.001).

**Fig. 3:**
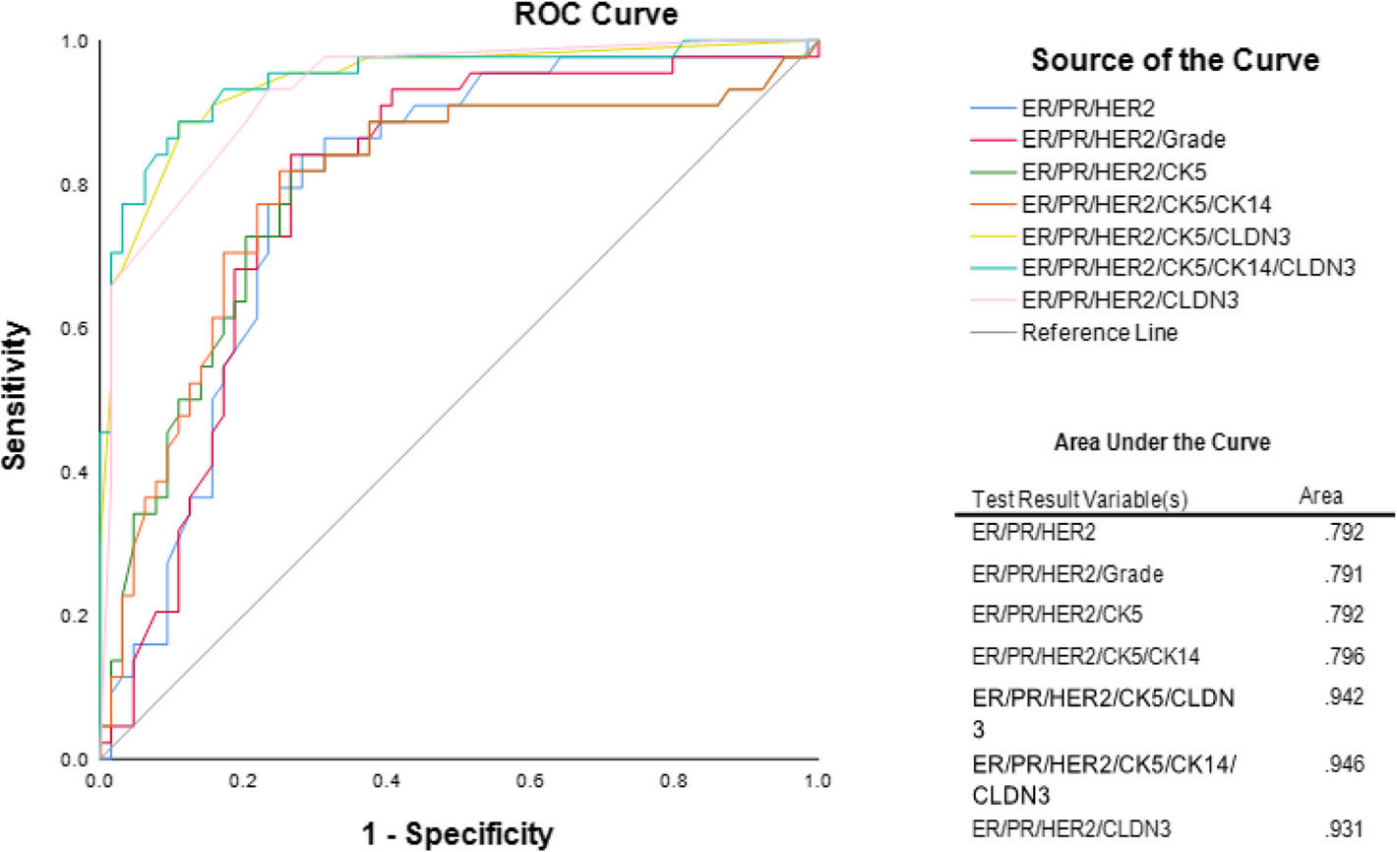
ROC curve for the discrimination between *BRCA1*-mutated tumors and *BRCA* WT tumors

Our receiver operation characteristic (ROC) analysis showed that the base model that correlates with *BRCA1* status, which consisted of only ER, PR and HER2, yielded an area under the curve (AUC) of 0.792. The addition of CLDN3 to this model resulted in an AUC of 0.931. When CK5 was added to this model, the model yielded an AUC of 0.942. Further addition of CK14 resulted in an AUC of 0.946 in the ROC curve which is shown in Fig. 3.

## Discussion

Taken together, results from our study showed that CLDN3, CK5, and CK14 are associated with *BRCA1* mutation status when used in a model where ER, PR and HER2 status are already known. Our research shows that staining of CLDN3, CK5, and CK14 in combination with ER, PR and HER2 indicate an association with *BRCA1* mutation status.

Our findings show that tumors with expression of CK5 and CK14 were more likely to arise in *BRCA1* than in the control group. This has been reported previously by Lakhani et al. where they investigated immunohistochemical staining for basal markers. They found that CK5/6, CK14, and EGFR were more frequent in *BRCA1* tumors compared to non-mutation BC (58% versus 7, 61% versus 12, 67% versus 21%, *p* < 0.0001 in each case, respectively) [42]. This supports that most of *BRCA1*-related cancers belong to the basal-like subtype [2]. Foulkes and colleagues showed that the expression of CK5/6 was statistically significantly associated with *BRCA1*-related BC [18, 50]. Furthermore, an association between positive CK5/6 and *BRCA* mutation status was shown by Murria Estal et al. [51] However, Mohanty et al. could not show a statistically significant difference of CK14 expression between *BRCA*-mutated and sporadic BC [52]. In addition, CK5/6, CK14, and E-cadherin were not associated with *BRCA1* status in a study from Hassanein et al. [53] Eerola et al. reported that CK14 was significantly associated with *BRCA1* tumors in univariate analysis [54]. In our study, the model which consisted of ER, PR, HER2, positive expression of CLDN3, CK5, and CK14 yielded an AUC of 0.946 in the ROC analysis to indicate an association with the *BRCA1* mutation status. We also found a significantly higher proportion of CK5 expression in *BRCA1* than *BRCA2* BC. EGFR, an additional biomarker of the basal-like subtype, negatively correlated with the *BRCA2* mutation status in comparison with *BRCA* WT in our study. Expression of CK5/6 and CK14 in *BRCA2*-related BC is rare and does not differ from sporadic tumors [7, 40–42, 55]. Results from our study showed similar findings. Otherwise, Eerola et al. showed a positive expression of CK5/6 and CK14 in 7.7 and 26.9%, respectively, in *BRCA2* tumors. Only regarding CK14, there was a significant difference between these tumors and the sporadic ones [54].

CLDN3 and CLDN4 are commonly expressed in BC [32, 56, 57]. Madaras et al. examined the expression of CLDN3, 4, and 7, among other variables, in *BRCA*-mutated and *BRCA* WT tumor tissues. In *BRCA*-mutated tumors, CLDN3, 4, and 7 were expressed at higher level compared to *BRCA* WT tumors [58]. Higher overexpression rates for CLDN3, 4, and 7 were found in *BRCA1*-related BC compared to sporadic BC [48]. We showed that CLDN3 was positively correlated with *BRCA1*-mutated BC. Positive CLDN3 was also significantly more frequent in the *BRCA2* in comparison with the WT group. However, in our study there was no association between CLDN4 or CLDN7 and *BRCA*-mutated BC found. Positive CLDN4 was very common, but CLDN7 was rarely positive in all groups (*BRCA1*, *BRCA2*, WT).

In the analysis of CIMBA (Consortium of Investigators of Modifiers of *BRCA1/2*), Mavaddat et al. showed that 78% of *BRCA1*-related tumors were ER-negative, but in patients with *BRCA2* mutation status ER was negative only in 23% of the tumors. The authors could demonstrate that in the *BRCA1* group, 79% were PR-negative and 90% HER2-negative compared to 36 and 87% in BC patients with *BRCA2* mutation status, respectively. These results allow to conclude that ER-positive tumors occur more likely in *BRCA2* than in *BRCA1* cases [44]. Several studies could also demonstrate that negative ER and PR were significantly more common among *BRCA1*-mutated tumors compared to sporadic cases, among others [11–13, 20, 48, 53]. Therefore, these results are similar to our findings. A low prevalence of positive HER2 (6.8%) expression was shown among patients with *BRCA2*-related BC by Evans et al., too [59]. *BRCA2* tumors often show no or low expression of HER2 [7, 13, 17, 40] However, Armes et al. showed a strong expression of HER2 in 44% (4 of 9) of *BRCA2* tumors [60]. Accordingly in our study, positive HER2 was found to be associated with a *BRCA2* mutation compared to the WT.

The limitations of this study are a small sample size resulting in a large OR as observed in the results for CLDN3, and should consequently be interpreted with caution. A larger sample size for future studies would be necessary to confirm these findings. Moreover, we did not analyze different molecular subtypes in this study. Future studies should therefore investigate the relationship between the molecular subtypes and the *BRCA* mutation status. Additionally, the problem of selection should be mentioned as a further limitation of our study. We selected patients with familial breast cancer based on the *BRCA1/2* mutation status. Thus, the controls consist of predominantly luminal subtypes.

The dataset of this study should be considered as a training dataset, the analysis is hypothesis generating. This aspect is one of the limitations of our study which is exploratory. We do not have a sufficiently high number of independently collected samples to test the parameters in an independent dataset, too. Thus, further studies are necessary regarding this point.

The examination of TMA should be mentioned as a further limitation of this study. However, the staining was fairly homogenous across the tumor. There were no areas with stronger or weaker intensity observed. TMA is an acceptable and sufficient device, which has been developed and used in research settings [61–65].

In conclusion, findings from our study showed that CLDN3, CK5, and CK14 in combination with ER, PR and HER2 are associated with *BRCA1* mutation status.

## Conclusions

Our data suggests that CLDN3, CK5, and CK14 in combination with ER, PR and HER2 indicate an association with *BRCA1* mutation status.

## Additional files

Additional file 1: BRCA testing and analysis (DOCX 16 kb)

Additional file 2: Data (XLSX 40 kb)

Additional file 3: Data dictionary (DOCX 21 kb)

## Data Availability

The datasets used and analyzed during the current study are available from the corresponding author on reasonable request. [Additional supporting files: “Data” (Additional file 2), “Data dictionary” (Additional file 3)]

## Abbreviations

AUC: Area under the curve
BC: Breast cancer
CI: Confidence interval
CK14: Cytokeratin 14
CK5: Cytokeratin 5
CLDN: Claudin
EGFR: Epidermal growth factor receptor
ER: Estrogen receptor
HER2: Human epidermal growth factor receptor 2
OR: Odds ratio
PR: Progesterone receptor
ROC: Receiver operation characteristic
TMA: Tissue microarray
WT: Wild type

## References

[1] Siegel RL, Miller KD, Jemal A (2016). Cancer statistics, 2016. CA Cancer J Clin.

[2] Melchor L, Benitez J (2013). The complex genetic landscape of familial breast cancer. Hum Genet.

[3] Miki Y, Swensen J, Shattuck-Eidens D, Futreal PA, Harshman K, Tavtigian S, Liu Q, Cochran C, Bennett LM, Ding W (1994). A strong candidate for the breast and ovarian cancer susceptibility gene BRCA1. Science.

[4] Wooster R, Neuhausen SL, Mangion J, Quirk Y, Ford D, Collins N, Nguyen K, Seal S, Tran T, Averill D (1994). Localization of a breast cancer susceptibility gene, BRCA2, to chromosome 13q12-13. Science.

[5] Antoniou A, Pharoah PD, Narod S, Risch HA, Eyfjord JE, Hopper JL, Loman N, Olsson H, Johannsson O, Borg A (2003). Average risks of breast and ovarian cancer associated with BRCA1 or BRCA2 mutations detected in case series unselected for family history: a combined analysis of 22 studies. Am J Hum Genet.

[6] Chen S, Parmigiani G (2007). Meta-analysis of BRCA1 and BRCA2 penetrance. J Clin Oncol.

[7] van der Groep P, van der Wall E, van Diest PJ (2011). Pathology of hereditary breast cancer. Cell Oncol (Dordr).

[8] Stratton Michael R (1997). Pathology of familial breast cancer: differences between breast cancers in carriers of BRCA1 or BRCA2 mutations and sporadic cases. The Lancet.

[9] Lakhani SR, Jacquemier J, Sloane JP, Gusterson BA, Anderson TJ, van de Vijver MJ, Farid LM, Venter D, Antoniou A, Storfer-Isser A (1998). Multifactorial analysis of differences between sporadic breast cancers and cancers involving BRCA1 and BRCA2 mutations. J Natl Cancer Inst.

[10] Honrado E, Benitez J, Palacios J (2005). The molecular pathology of hereditary breast cancer: genetic testing and therapeutic implications. Mod Pathol.

[11] Eerola H, Heikkila P, Tamminen A, Aittomaki K, Blomqvist C, Nevanlinna H (2005). Histopathological features of breast tumours in BRCA1, BRCA2 and mutation-negative breast cancer families. Breast Cancer Res.

[12] Chappuis PO, Nethercot V, Foulkes WD (2000). Clinico-pathological characteristics of BRCA1- and BRCA2-related breast cancer. Semin Surg Oncol.

[13] Palacios J, Honrado E, Osorio A, Cazorla A, Sarrio D, Barroso A, Rodriguez S, Cigudosa JC, Diez O, Alonso C (2003). Immunohistochemical characteristics defined by tissue microarray of hereditary breast cancer not attributable to BRCA1 or BRCA2 mutations: differences from breast carcinomas arising in BRCA1 and BRCA2 mutation carriers. Clin Cancer Res.

[14] Rakha EA, Reis-Filho JS, Ellis IO (2008). Basal-like breast cancer: a critical review. J Clin Oncol.

[15] Reis-Filho JS, Tutt AN (2008). Triple negative tumours: a critical review. Histopathology.

[16] Lachapelle J, Foulkes WD (2011). Triple-negative and basal-like breast cancer: implications for oncologists. Curr Oncol.

[17] Lakhani SR, Van De Vijver MJ, Jacquemier J, Anderson TJ, Osin PP, McGuffog L, Easton DF (2002). The pathology of familial breast cancer: predictive value of immunohistochemical markers estrogen receptor, progesterone receptor, HER-2, and p53 in patients with mutations in BRCA1 and BRCA2. J Clin Oncol.

[18] Foulkes WD, Stefansson IM, Chappuis PO, Begin LR, Goffin JR, Wong N, Trudel M, Akslen LA (2003). Germline BRCA1 mutations and a basal epithelial phenotype in breast cancer. J Natl Cancer Inst.

[19] Rodriguez-Pinilla SM, Sarrio D, Honrado E, Moreno-Bueno G, Hardisson D, Calero F, Benitez J, Palacios J (2007). Vimentin and laminin expression is associated with basal-like phenotype in both sporadic and BRCA1-associated breast carcinomas. J Clin Pathol.

[20] Triantafyllidou O, Vlachos IS, Apostolou P, Konstantopoulou I, Grivas A, Panopoulos C, Dimitrakakis C, Kassanos D, Loghis C, Bramis I (2015). Epidemiological and clinicopathological characteristics of BRCA-positive and BRCA-negative breast cancer patients in Greece. J BUON.

[21] Foulkes WD, Smith IE, Reis-Filho JS (2010). Triple-negative breast cancer. N Engl J Med.

[22] Nielsen TO, Hsu FD, Jensen K, Cheang M, Karaca G, Hu Z, Hernandez-Boussard T, Livasy C, Cowan D, Dressler L (2004). Immunohistochemical and clinical characterization of the basal-like subtype of invasive breast carcinoma. Clin Cancer Res.

[23] Cheang MC, Voduc D, Bajdik C, Leung S, McKinney S, Chia SK, Perou CM, Nielsen TO (2008). Basal-like breast cancer defined by five biomarkers has superior prognostic value than triple-negative phenotype. Clin Cancer Res.

[24] Perou CM, Sorlie T, Eisen MB, van de Rijn M, Jeffrey SS, Rees CA, Pollack JR, Ross DT, Johnsen H, Akslen LA (2000). Molecular portraits of human breast tumours. Nature.

[25] Sorlie T, Perou CM, Tibshirani R, Aas T, Geisler S, Johnsen H, Hastie T, Eisen MB, van de Rijn M, Jeffrey SS (2001). Gene expression patterns of breast carcinomas distinguish tumor subclasses with clinical implications. Proc Natl Acad Sci U S A.

[26] Carey LA, Perou CM, Livasy CA, Dressler LG, Cowan D, Conway K, Karaca G, Troester MA, Tse CK, Edmiston S (2006). Race, breast cancer subtypes, and survival in the Carolina breast Cancer study. JAMA.

[27] Tang P, Skinner KA, Hicks DG (2009). Molecular classification of breast carcinomas by immunohistochemical analysis: are we ready?. Diagn Mol Pathol.

[28] Badve S, Dabbs DJ, Schnitt SJ, Baehner FL, Decker T, Eusebi V, Fox SB, Ichihara S, Jacquemier J, Lakhani SR (2011). Basal-like and triple-negative breast cancers: a critical review with an emphasis on the implications for pathologists and oncologists. Mod Pathol.

[29] Herschkowitz JI, Simin K, Weigman VJ, Mikaelian I, Usary J, Hu Z, Rasmussen KE, Jones LP, Assefnia S, Chandrasekharan S (2007). Identification of conserved gene expression features between murine mammary carcinoma models and human breast tumors. Genome Biol.

[30] Prat A, Parker JS, Karginova O, Fan C, Livasy C, Herschkowitz JI, He X, Perou CM (2010). Phenotypic and molecular characterization of the claudin-low intrinsic subtype of breast cancer. Breast Cancer Res.

[31] Perou CM (2011). Molecular stratification of triple-negative breast cancers. Oncologist.

[32] Ding L, Lu Z, Lu Q, Chen YH (2013). The claudin family of proteins in human malignancy: a clinical perspective. Cancer Manag Res.

[33] Lu S, Singh K, Mangray S, Tavares R, Noble L, Resnick MB, Yakirevich E (2013). Claudin expression in high-grade invasive ductal carcinoma of the breast: correlation with the molecular subtype. Mod Pathol.

[34] van Roy F, Berx G (2008). The cell-cell adhesion molecule E-cadherin. Cell Mol Life Sci.

[35] Strumane K, Berx G, Van Roy F (2004). Cadherins in cancer. Handb Exp Pharmacol.

[36] David JM, Rajasekaran AK (2012). Dishonorable discharge: the oncogenic roles of cleaved E-cadherin fragments. Cancer Res.

[37] Frixen UH, Behrens J, Sachs M, Eberle G, Voss B, Warda A, Lochner D, Birchmeier W (1991). E-cadherin-mediated cell-cell adhesion prevents invasiveness of human carcinoma cells. J Cell Biol.

[38] Vleminckx K, Vakaet L, Mareel M, Fiers W, van Roy F (1991). Genetic manipulation of E-cadherin expression by epithelial tumor cells reveals an invasion suppressor role. Cell.

[39] Perl AK, Wilgenbus P, Dahl U, Semb H, Christofori G (1998). A causal role for E-cadherin in the transition from adenoma to carcinoma. Nature.

[40] Bane AL, Beck JC, Bleiweiss I, Buys SS, Catalano E, Daly MB, Giles G, Godwin AK, Hibshoosh H, Hopper JL (2007). BRCA2 mutation-associated breast cancers exhibit a distinguishing phenotype based on morphology and molecular profiles from tissue microarrays. Am J Surg Pathol.

[41] Palacios J, Honrado E, Osorio A, Cazorla A, Sarrio D, Barroso A, Rodriguez S, Cigudosa JC, Diez O, Alonso C (2005). Phenotypic characterization of BRCA1 and BRCA2 tumors based in a tissue microarray study with 37 immunohistochemical markers. Breast Cancer Res Treat.

[42] Lakhani SR, Reis-Filho JS, Fulford L, Penault-Llorca F, van der Vijver M, Parry S, Bishop T, Benitez J, Rivas C, Bignon YJ (2005). Prediction of BRCA1 status in patients with breast cancer using estrogen receptor and basal phenotype. Clin Cancer Res.

[43] Larsen MJ, Thomassen M, Gerdes AM, Kruse TA (2014). Hereditary breast cancer: clinical, pathological and molecular characteristics. Breast Cancer (Auckl).

[44] Mavaddat N, Barrowdale D, Andrulis IL, Domchek SM, Eccles D, Nevanlinna H, Ramus SJ, Spurdle A, Robson M, Sherman M (2012). Pathology of breast and ovarian cancers among BRCA1 and BRCA2 mutation carriers: results from the consortium of investigators of modifiers of BRCA1/2 (CIMBA). Cancer Epidemiol Biomark Prev.

[45] Danzinger S, Tan YY, Rudas M, Kastner MT, Weingartshofer S, Muhr D, Singer CF (2018). kConFab I: differential Claudin 3 and EGFR expression predicts BRCA1 mutation in triple-negative breast Cancer. Cancer Investig.

[46] Hammond ME, Hayes DF, Dowsett M, Allred DC, Hagerty KL, Badve S, Fitzgibbons PL, Francis G, Goldstein NS, Hayes M (2010). American Society of Clinical Oncology/College of American Pathologists guideline recommendations for immunohistochemical testing of estrogen and progesterone receptors in breast cancer. J Clin Oncol.

[47] Wolff AC, Hammond ME, Hicks DG, Dowsett M, McShane LM, Allison KH, Allred DC, Bartlett JM, Bilous M, Fitzgibbons P (2014). Recommendations for human epidermal growth factor receptor 2 testing in breast cancer: American Society of Clinical Oncology/College of American Pathologists clinical practice guideline update. Arch Pathol Lab Med.

[48] Heerma van Voss MR, van Diest PJ, Smolders YH, Bart J, van der Wall E, van der Groep P (2014). Distinct claudin expression characterizes BRCA1-related breast cancer. Histopathology.

[49] Christgen M, Noskowicz M, Schipper E, Christgen H, Heil C, Krech T, Langer F, Kreipe H, Lehmann U (2013). Oncogenic PIK3CA mutations in lobular breast cancer progression. Genes Chromosomes Cancer.

[50] Palacios J, Honrado E, Osorio A, Diez O, Rivas C, Benitez J (2004). Re: Germline BRCA1 mutations and a basal epithelial phenotype in breast cancer. J Natl Cancer Inst.

[51] Murria Estal R, Palanca Suela S, de Juan JI, Alenda Gonzalez C, Egoavil Rojas C, Garcia-Casado Z, Lopez Guerrero JA, Juan Fita MJ, Sanchez Heras AB, Segura Huerta A (2016). Relationship of immunohistochemistry, copy number aberrations and epigenetic disorders with BRCAness pattern in hereditary and sporadic breast cancer. Familial Cancer.

[52] Mohanty SK, Lai JP, Gordon OK, Pradhan D, Bose S, Dadmanesh F (2015). BRCA-mutated invasive breast carcinomas: Immunohistochemical analysis of insulin-like growth factor II mRNA-binding protein (IMP3), cytokeratin 8/18, and cytokeratin 14. Breast J.

[53] Hassanein M, Huiart L, Bourdon V, Rabayrol L, Geneix J, Nogues C, Peyrat JP, Gesta P, Meynard P, Dreyfus H (2013). Prediction of BRCA1 germ-line mutation status in patients with breast cancer using histoprognosis grade, MS110, Lys27H3, vimentin, and KI67. Pathobiology.

[54] Eerola H, Heinonen M, Heikkila P, Kilpivaara O, Tamminen A, Aittomaki K, Blomqvist C, Ristimaki A, Nevanlinna H (2008). Basal cytokeratins in breast tumours among BRCA1, BRCA2 and mutation-negative breast cancer families. Breast Cancer Res.

[55] Honrado E, Osorio A, Palacios J, Milne RL, Sanchez L, Diez O, Cazorla A, Syrjakoski K, Huntsman D, Heikkila P (2005). Immunohistochemical expression of DNA repair proteins in familial breast cancer differentiate BRCA2-associated tumors. J Clin Oncol.

[56] Singh AB, Sharma A, Dhawan P (2010). Claudin family of proteins and cancer: an overview. J Oncol.

[57] Kominsky SL, Vali M, Korz D, Gabig TG, Weitzman SA, Argani P, Sukumar S (2004). Clostridium perfringens enterotoxin elicits rapid and specific cytolysis of breast carcinoma cells mediated through tight junction proteins claudin 3 and 4. Am J Pathol.

[58] Madaras L, Balint N, Gyorffy B, Tokes AM, Barshack I, Yosepovich A, Friedman E, Paluch-Shimon S, Zippel D, Baghy K (2016). BRCA mutation-related and Claudin-low breast Cancer: blood relatives or stepsisters. Pathobiology.

[59] Evans DG, Lalloo F, Howell S, Verhoef S, Woodward ER, Howell A (2016). Low prevalence of HER2 positivity amongst BRCA1 and BRCA2 mutation carriers and in primary BRCA screens. Breast Cancer Res Treat.

[60] Armes JE, Trute L, White D, Southey MC, Hammet F, Tesoriero A, Hutchins AM, Dite GS, McCredie MR, Giles GG (1999). Distinct molecular pathogeneses of early-onset breast cancers in BRCA1 and BRCA2 mutation carriers: a population-based study. Cancer Res.

[61] Kononen J, Bubendorf L, Kallioniemi A, Barlund M, Schraml P, Leighton S, Torhorst J, Mihatsch MJ, Sauter G, Kallioniemi OP (1998). Tissue microarrays for high-throughput molecular profiling of tumor specimens. Nat Med.

[62] Kyndi M, Sorensen FB, Knudsen H, Overgaard M, Nielsen HM, Andersen J, Overgaard J (2008). Tissue microarrays compared with whole sections and biochemical analyses. A subgroup analysis of DBCG 82 b&c. Acta Oncol.

[63] Rossing HH, Talman ML, Laenkholm AV, Wielenga VT (2012). Implementation of TMA and digitalization in routine diagnostics of breast pathology. APMIS.

[64] Dekker TJ, ter Borg S, Hooijer GK, Meijer SL, Wesseling J, Boers JE, Schuuring E, Bart J, van Gorp J, Bult P (2015). Quality assessment of estrogen receptor and progesterone receptor testing in breast cancer using a tissue microarray-based approach. Breast Cancer Res Treat.

[65] Dekker TJ, ter Borg S, Hooijer GK, Meijer SL, Wesseling J, Boers JE, Schuuring E, Bart J, van Gorp J, Bult P (2015). Erratum to: quality assessment of estrogen receptor and progesterone receptor testing in breast cancer using a tissue microarray-based approach. Breast Cancer Res Treat.

