# Supplementary material for: Association of Cytokeratin 5 and Claudin 3 expression with BRCA1 and BRCA2 germline mutations in women with early breast cancer
Source: BMC Cancer. 2019 Jul 15;19:695. doi: 10.1186/s12885-019-5908-6 (PMC6631579; doi:10.1186/s12885-019-5908-6)
Supplement: Supplementary file 1 — BRCA testing and analysis (DOCX 16 kb) [file 12885_2019_5908_MOESM1_ESM.docx]

**Additional file 1**

*BRCA* testing and analysis

The tissue microarrays (TMA) were obtained from the Kathleen Cuningham Foundation Consortium for research into Familial Breast cancer (kConFab) [<http://www.kconfab.org>], classified into the subgroups depending on the mutation status.

*BRCA* results were determined by sequence-based testing and the multiplex ligation-dependent probe amplification (MLPA) technique.

Originally, the mutation classification committee of kConFab used the BIC (Breast Cancer Information Core) database and publications/evidence for classification, these days the IARC (International Agency on Cancer Research) 5 tier classification system.
