# Supplementary material for: Association of Cytokeratin 5 and Claudin 3 expression with BRCA1 and BRCA2 germline mutations in women with early breast cancer
Source: BMC Cancer. 2019 Jul 15;19:695. doi: 10.1186/s12885-019-5908-6 (PMC6631579; doi:10.1186/s12885-019-5908-6)
Supplement: Supplementary file 3 — Data dictionary (DOCX 21 kb) [file 12885_2019_5908_MOESM3_ESM.docx]

**Data dictionary**

BRCA

BRCA1 mutation carrier 1

BRCA2 mutation carrier 2

no BRCA1-/2-mutation 0

Survival

alive … 0

deceased … 1

Morphology

Invasive ductal carcinoma … 1

Invasive lobular carcinoma … 2

Invasive ductal and lobular carcinoma … 3

Ductal carcinoma in situ … 4

Carcinoma – undefined … 5

Other … 6

not available … free

Tumor size

< 2 cm … 1

2-5 cm … 2

> 5 cm … 3

not available … free

Grade

Grade I … 1

Grade II … 2

Grade III … 3

not available … free

Estrogen receptor

positive … 1

negative … 0

not available … free

Progesterone receptor

positive … 1

negative … 0

not available … free

HER2

positive … 1

negative … 0

not available … free

CK 5

positive … 1

negative … 0

not available … free

CK 14

positive … 1

negative … 0

not available … free

EGFR

positive … 1

negative … 0

not available … free

Claudin 3

positive … 1

negative … 0

not available … free

Claudin 4

positive … 1

negative … 0

not available … free

Claudin 7

positive … 1

negative … 0

not available … free

E-cadherin

positive … 1

negative … 0

not available … free
